# Dysregulation of Cholesterol Homeostasis in Ovarian Cancer

**DOI:** 10.3390/curroncol30090609

**Published:** 2023-09-13

**Authors:** Zahraa Qusairy, Anne Gangloff, Shuk On Annie Leung

**Affiliations:** 1 Cancer Research Program, Research Institute of the McGill University Health Centre, Montreal, QC H4A 3J1, Canada; 2 CHU de Québec-Université Laval Research Center, Québec City, QC G1V 4G2, Canada; anne.gangloff.1@ulaval.ca; 3 Faculty of Medicine, Laval University, Québec City, QC G1V 0A6, Canada; 4 Division of Gynecologic Oncology, Department of Obstetrics and Gynecology, McGill University Health Centre, McGill University, Montreal, QC H4A 3J1, Canada

**Keywords:** cholesterol, ovarian cancer, cell proliferation, chemoresistance, metastases

## Abstract

Cholesterol plays an essential role in maintaining the rigidity of cell membranes and signal transduction. Various investigations confirmed empirically that the dysregulation of cholesterol homeostasis positively correlates with tumor progression. More specifically, recent studies suggested the distinct role of cholesterol in ovarian cancer cell proliferation, metastasis and chemoresistance. In this review, we summarize the current findings that suggest the contribution of cholesterol homeostasis dysregulation to ovarian cancer progression and resistance to anti-cancer agents. We also discuss the therapeutic implications of cholesterol-lowering drugs in ovarian cancer.

## 1. Introduction

Ovarian cancer (OC) is the fifth most lethal gynecologic malignancy and accounts for 4% of all cancer-related deaths in women [1]. The median 5-year relative survival rate for all patients with OC is 50.8% and ranges from 89% for patients diagnosed at stage I to 20% for patients diagnosed at stage IV [1,2]. Unfortunately, over 70% of OC are diagnosed at advanced stages and metastasis is common [3,4] Ovarian cancer is categorized into three main groups: epithelial, germ cell and sex-cord stromal tumors [2]. The epithelial type accounts for over 95% of the ovarian malignancies [5]. The epithelial type of OC is subdivided into five major histological subtypes, including high-grade serous (HGSOC), low-grade serous (LGSOC), endometrioid (ENOC), clear cell (CCOC) and mucinous (MOC) [6]. Each subtype has distinct cells of origin, molecular features, clinical features and treatments [3]. The dominant subtype is HGSOC, which accounts for approximately 75% of all OC [7].

As one of the main energy sources, lipids are involved in various extracellular and cellular biological functions, such as components of cell membranes, messengers and signaling molecules involved in generating and maintaining the biological functions of the body [8,9,10,11]. Cholesterol is a lipophilic molecule and an important constituent of the lipid fraction of the human body. Cholesterol is precursor to three classes of molecules: steroid hormones, vitamin D and its derivatives, as well as bile acids [12]. Due to its lack of solubility, cholesterol is transported through the blood using lipoprotein particles carriers that shuttle cholesterol and lipids throughout the body. Dietary cholesterol is transported in chylomicrons while the liver secretes very-low-density lipoproteins (VLDL) that will be metabolized to intermediate-density lipoproteins (IDL) and low-density lipoproteins (LDL), the latter providing cholesterol to peripheral cells. High-density lipoproteins (HDL) are lipoproteins that transport cholesterol from the periphery to the liver, among other roles [13]. Of note, a major proportion of cholesterol is transported via LDL and VLDL [14]. There is emerging evidence which suggests a significant association between cholesterol levels and cancer [11,15,16,17]. Levels of serum cholesterol are associated with a higher risk of cancer cell proliferation, metastasis and resistance to anti-cancer agents [11,17,18,19]. Therefore, an increasing number of investigations focus on the potential of repurposing statins, drugs which decrease cholesterol, as anti-cancer agents in OC (e.g., NCT04457089 which investigated simvastatin 40 mg in OC, NCT03532139 which investigated rosuvastatin and enoxaparin following OC surgery, and NCT00585052 which investigated lovastatin and paclitaxel in recurrent OC) [20,21,22].

OC is among the cancers that are highly impacted by serum cholesterol levels [23]. Cholesterol metabolites, such as 27-hydroxycholesterol (27HC), have also been shown to induce ovarian cancer progression [17]. Zheng et al. have identified the upregulation of sterol regulatory element binding transcription factor 2 (SREBF2), a master regulator of cholesterol synthesis, in cisplatin-resistant ovarian cancer cells [24]. Clinical monitoring of lipid profile has also been suggested as a method of early diagnosis based on the observation of a dramatic drop in HDL cholesterol in the year prior OC diagnosis [25]. Furthermore, Visvanathan et al. reported a 43% reduction in mortality with the use of lipophilic statins (simvastatin and atorvastatin) based on an analysis of over 10,000 cases of OC in the Finnish National Cancer Registry [26]. In this review, we first discuss the role of cholesterol and its metabolites in OC, as well as the potential signaling pathways and molecules involved in increasing the levels of cholesterol in serum, followed by discussing the potential utility of hypocholesterolemic drugs.

## 2. Physiology of Cholesterol Metabolism

The two main sources of cholesterol are dietary (exogenous) and de novo, mainly by, the liver but also by adrenal gland, intestine, and gonads [27]. In the healthy state, exogenous cholesterol absorption is mediated by the Niemann–Pick C1-like-1 (NPC1L1) protein, which facilitates cholesterol uptake from intestinal epithelial cells [28]. Following absorption, cholesterol is incorporated into chylomicrons, which are lipoproteins produced by enterocytes in the intestine Chylomicrons, have an apo B-48 protein as structural protein, and can bear other lipoproteins at their surface such as apo C-II and apo A-I [29,30], Chylomicrons deliver triglycerides to extrahepatic tissues through lipoprotein lipase hydrolysis. Triglycerides depleted chylomicrons are called chylomicron remnants and must acquire an apo E to be cleared from the blood stream through uptake by the liver apo B/E Receptor (also known as LRP1) [31].

In the de novo pathway, synthesis begins with the conversion of mitochondrial acetyl-CoA from hepatic mitochondria into 3-hydroxy-3-methylglutaryl (HMG)-CoA by 3-hydroxy-3-methylglutaryl-CoA synthase (HMGCS) and HMG-CoA reductase (HMGCR) [32]. This enzyme is the rate-limiting step of cholesterol biosynthesis and is the target of statins used commonly to decrease cholesterol. Through phosphorylation reactions, HMG-CoA is converted into squalene, lanosterol and finally cholesterol [33]. Cholesterol being lipophilic, the liver packages cholesterol into VLDL which are then secreted into the blood stream where they will deliver lipids to peripheral tissues. VLDL have apo B-100 as their core protein but can gain and/or exchange a variety of different apolipoproteins while in the blood stream, such as apo C-II, apo CIII, apo E, among others. VLDL are hydrolyzed into IDL then LDL by lipoprotein lipase, a process which permits to supply fatty acids to cells. Peripheral cells can either produce their own cholesterol or take up beta-lipoproteins through apo B/E receptors. Cholesterol is used by peripheral tissues as a component of cell membranes for the production of steroids hormones, vitamin D and its derivatives, as well as bile salts [34]. HDL particles are produced by the liver and the enterocytes and gain lipids from peripheral cells. The fate of HDL (which contains apolipoproteins apo A-I, and apo A-II) is either recycled for other tissues in need of cholesterol or returned to the liver to be excreted in a process named “reverse cholesterol transport” [29,30]. Excess peripheral cholesterol can also be converted into cholesteryl ester through acetyltransferase ACAT1 and stored as lipid droplets intracellularly [28].

The balance of this complex process of cholesterol metabolism is maintained through the “sterol-sensitive system” [28]. First, in response to decreased sterol levels, SREBP2, located in the endoplasmic reticulum (ER), which is normally bound to SREBP-cleavage activating protein (SCAP), dissociates and releases SREBP2/SCAP via coatomer protein II (COPII) vesicles to relocate it to the Golgi apparatus [35]. In the Golgi apparatus, SEBP2/SCAP is cleaved by membrane-bound proteases, site-1 protease (S1P) and site-2 protease (S2P), releasing the active form, nuclear SREBP2 (nSREBP2). The binding of nSREBP2 to the sterol response elements of the HMGCR and SQLE genes initiates transcription, resulting in intracellular cholesterol synthesis [36]. Moreover, the activation of SREBP2 induces the absorption and uptake of cholesterol by increasing the expression of NPC1L1 and LDL receptors respectively [36]. Alternatively, when the peripheral cholesterol levels are high, liver X receptors (LXRs), which are activated by forming a heterodimer complex with retinoid X receptor-α (RXR α), trigger reverse cholesterol transport from the peripheral tissues by increasing synthesis of hepatic Apo A1 and secretion of nascent pre-beta HDL [28,32]. Nascent discoid HDL secreted by the liver and by enterocytes acquire cholesterol and other lipides by contact with tissues, a process mediated by ABCA1 transporters (cellular cholesterol efflux) and by LCAT (cholesterol esterification), to become spherical, mature HDL [37]. In addition to their role in the cholesterol efflux metabolism, LXRs are also involved in the conversion of cholesterol into bile acids by cholesterol 7α-hydroxylase (CYP7A1), as well as inhibiting the absorption and uptake of exogenous cholesterol by decreasing the expression rates of the NPC1L1 and LDL receptors [28,38]. Lastly, nuclear factor erythroid 2-related factor-1 (NRF1), located in the ER, binds directly to the cholesterol molecules, causing a series of modifications to discard excessive amount of intracellular cholesterol that damage cells, especially hepatocytes [39].

## 3. Cholesterol Metabolism Dysregulation in Cancer

Aberrant cholesterol metabolism has been proposed as a metabolic hallmark in various cancers, including ovarian cancer [23]. Given the high cholesterol requirements of rapidly dividing cells, cancer cells rewire cholesterol metabolism pathways to secure abundant supplies of cholesterol and its metabolites [28]. This disturbance in cholesterol homeostasis is achieved by blocking sensor genes to high cholesterol levels and upregulating the genes and enzymes that are involved in the production and uptake of cholesterol [11,28,40]. An enzyme upregulated by cancer cells is squalene synthase (SQS), which mediates cholesterol synthesis and the buildup of tumor necrosis factor receptor 1 (TNFR1) in the lipid rafts, can induce either apoptosis or proliferation depending on the environmental context, as well as NF-κB and MAP kinase activation [28,41]. 2,3-oxidosqualene cyclase (OSC) referred to as lanosterol synthase (LSS) [42], mediates cancer neovascularization and metastasis through the activation of the PI3K/Akt signaling pathway and FGF-2 [28,43]. The oncogene c-Myc (MYC) promotes cell growth and proliferation as well as induces the expression of HMGCR [44,45].

The aberrant expression of epidermal growth factor activates SCAP- mediated SREBP-2 activation, leading to elevated LDLR expression and consequently the cellular uptake of cholesterol [28,46,47]. The c-fos proto-oncogene decreases the expression of LXRs, which results in a rise in cholesterol levels [48]. Therefore, cancer cells disturb the intracellular cholesterol homeostasis, resulting in an altered cell membrane composition and cholesterol storage. Luchetti et al. [49] elucidated the role of cholesterol in the activation of smoothened (SMO), a G-protein-coupled receptor (GPCR) involved in the activation of the Hedgehog signaling pathway, an important developmental pathway that regulates cell growth, differentiation, and tissue patterning; the aberrant activation of this pathway contributes to tumor growth and progression.

### 3.1. The Role of Aberrant Cholesterol Metabolism in Ovarian Cancer Proliferation

In ovarian cancer, dysregulated lipid metabolism has been associated with cancer cell proliferation, tumor progression, metastasis and resistance against anti-cancer agents [8,50]. It is well established that the tumor microenvironment (TME) plays an essential role in tumorigenesis, tumor immune escape, chemoresistance, metastasis and recurrence [51]. The TME in ovarian cancer is a lipid-rich milieu due to the propensity of the tumor to metastasize to the omentum, a layer of adipose tissue that covers the intra-peritoneal organs [52] and is characterized by an insufficient supply of glucose and oxygen [23]. In order to overcome the anaerobic and hypoglycemic conditions, tumor cells utilize reprogrammed adaptive metabolism and alter the lipid metabolism [23]. This adaptation results in cancer cell proliferation, metastasis, and drug resistance [53,54].

The alteration of cholesterol metabolism has been shown to induce cell proliferation in ovarian cancer [23,55]. The molecular mechanisms underlying cholesterol-induced cell proliferation in ovarian cancer are an area of active investigation. For example, the knockdown of cholesterol synthesis genes, such as SREBP2 and FDFT1, in A2780 ovarian cancer cells significantly attenuated cell proliferation [24]. Cholesterol metabolites such as 27HC have been shown to play a pivotal role in cell proliferation in ovarian cancer. The knockout of host CYP27A1, the enzyme responsible for 27HC synthesis, significantly impaired the development of ovarian cancer in vivo [24]. A pathway that is of particular interest is the phosphoinositide 3 kinase (PI3K)/mammalian (or mechanistic) target of rapamycin (mTOR) pathway [56,57]. It has been reported that high levels of cholesterol synthesis result in the upregulation of acetyl-coenzyme A acetyl transferase 1 (ACAT1), which induces cancer cell proliferation through the activation of the PI3K/mTOR pathway [56,58]. ACAT1 levels in the peritoneal fluid and tumor tissue are significantly higher before resection in ovarian cancer patients [59]. Cholesterol synthesis is also shown to inhibit phosphatase and tensin (PTEN), which is known as a negative regulator of the PI3K/mTOR pathway [10,60]. Moreover, treating cancer cells with cholesterol-lowering drugs including simvastatin significantly attenuated cell cancer cell proliferation via suppression of the PI3K/mTOR pathway [61]. Of note, simvastatin is one of the many inhibitors of HMG-CoA reductase, the rate-limiting step of the mevalonate pathway [61]. Therefore, high levels of cholesterol may facilitate cancer cell proliferation through the upregulation of the PI3K/mTOR pathway in ovarian cancer.

### 3.2. The Role of Aberrant Cholesterol Metabolism in Ovarian Cancer Metastasis

Various studies have suggested that aberrant lipid metabolism including cholesterol metabolism positively correlates with metastasis in OC [23,62,63]. Fluidity versus membrane rigidity in metastatic cancer cells is governed by the production of lipid rafts packed in the liquid phase order to facilitates a variety of cellular signaling pathways that are interconnected with cancer-related processes [53,64]. CD36 plays a key role in cholesterol absorption, synthesis and transport [65]. Lengyel et al. analyzed the expression of CD36 in a matched cohort of primary and metastatic ovarian tumors, and the result suggested the upregulation of CD36 in metastatic tumors [66]. Moreover, the knockdown of CD36 in OC cells dramatically downregulated their capacity for invasion, adhesion and peritoneal dissemination [66]. These results further confirmed the use of anti-CD36 monoclonal antibodies in ovarian cancer mouse xenografts, in which treatment with anti-CD36 antibodies diminished the tumor burden [66]. Proprotein convertase subtilisin/kexin type 9 (PCSK9) is one of the main enzymes that regulates cholesterol homeostasis, inducing the degradation of LDL receptors, thereby reducing the clearance of LDL cholesterol [67]. Importantly, the ectopic overexpression of PCSK9 in JHOS2 ovarian cancer cells significantly induced the expression of AKT/MEK/ERK signaling [68], which plays an essential role in metastasis in OC [69]. Other studies suggested that the inhibition of cholesterol synthesis via statin, an inhibitor of HMG-CoA, impairs metastasis in ovarian cancer cells [22]. The results of the abovementioned studies support an important role of cholesterol metabolism on OC metastasis and the use of hypocholesterolemic agents may potentially be an effective treatment strategy against metastasis in ovarian cancer.

### 3.3. The Role of Aberrant Cholesterol Metabolism in Ovarian Cancer Drug Resistance

Accumulating evidence suggests the involvement of aberrant lipid metabolism in inducing resistance to anti-cancer drugs [23]. For example, by downregulating ABCA1 expression, involved in the efflux of cholesterol from cells, cholesterol is accumulated within cells and the integration of cholesterol into the cell membrane lowers permeability, which contributes to resistance development [53,64]. Kim et al. have shown that elevated cholesterol levels in ascites correlate with the chemoresistance of cancer cells against cisplatin and paclitaxel [70]. Accordingly, high levels of cholesterol stimulate the expression levels of drug efflux pump proteins, including ABCG2 and MDR1 [70]. It is worth mentioning that the proteins involved in cholesterol uptake are upregulated in chemo-resistant OC cells. In this context, the expression of LDL receptor (LDLR), the primary mediator of extracellular cholesterol uptake, was significantly upregulated in platinum-resistant OC cells (PEA2 and PEO23) compared to their sensitive counterparts, PEA1 and PEO14 [71]. Interestingly, the majority of the collected cholesterol was accumulated in the cell membranes of cisplatin-resistant cells [71].

SREBP2 is a transcription factor that mainly regulates the expression of the genes encoding enzymes associated with cholesterol metabolism, such as LDLR, FDFT1 and HMGCR [35]. Zheng et al. have suggested the contribution of SREBP2 to cisplatin resistance in OC, as they noticed its upregulation in cisplatin-resistant A2780 OC cells compared to their cisplatin-sensitive counterparts [24]. The expression levels of SREBP2 and its target genes, including LDLR and FDFT1, were significantly elevated upon exposing A2780 cells to cisplatin treatment, indicating that cisplatin may enhance SREBP2 expression [24]. Furthermore, the knockdown of SREBP2 increased the efficacy of cisplatin against A2780 cells [24]. In parallel, other studies have found a positive correlation between LDLR expression and cisplatin resistance in OC, while it is negatively associated with the overall survival rate [72]. LDLR is regulated by PCSK9, which binds to LDLR and thus triggers its breakdown [11,67].

Lastly, emerging studies have suggested the involvement of cholesterol metabolites in ovarian cancer resistance [23]. The upregulation of 27HC, a primary metabolite of cholesterol, has been linked to drug resistance to carboplatin in ovarian cancer cells [73]. Taken together, these data suggest a positive correlation between dysregulated cholesterol metabolism and anti-cancer drug resistance in OC.

## 4. Potential Target Pathways in Cholesterol Metabolism

**Figure 1:**
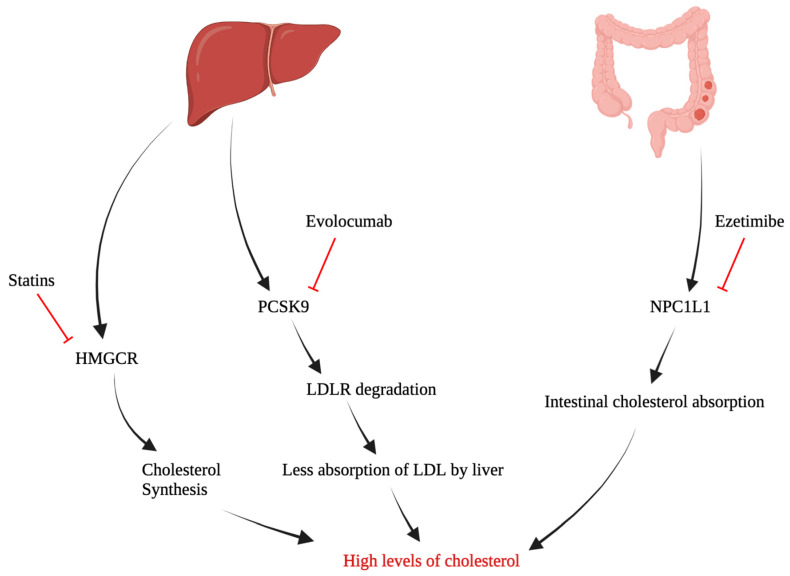
Mechanistic pathways underlying high levels of cholesterol and their blocking drugs.

The alteration of cholesterol metabolism is associated with different signaling pathways, such as cholesterol biosynthesis and cholesterol uptake (Figure 1). One of the main sources of cholesterol is cholesterol biosynthesis [74]. The biosynthesis of cholesterol mainly takes place in the liver and small intestine, starting from two molecules of acetyl-COA and a cascade of enzymatic reactions that depends on SREBP2 activation [29,75]. As mentioned above, SREBP2 is upregulated in OC cells with high levels of cell proliferation and resistance to cisplatin [24]. SREBP2 has various target genes that are intensely involved in cholesterol metabolism, including HMGCR [76,77]. HMGCR protein converts 3-hydroxy-3-methylglutaryl-CoA into mevalonate, which undergoes a series of enzymatic reactions, ending with a cholesterol molecule [78]. Interestingly, previous studies have demonstrated the upregulation of HMGCR in tumor cells [79]. Xia et al. [20] have shown the significant upregulation of HMGCR in OC cells, specifically those with a TP53 mutation. Of note, mutations of TP53 are among the most common mutations in ovarian cancer [80]. Another biosynthetic pathway within the liver is through PCSK9, which binds to LDLR, triggering its breakdown and decreasing the absorption of LDL by the liver, resulting in higher peripheral levels of cholesterol [11].

Alternatively, since cholesterol biosynthesis requires high levels of energy and precursors, cancer cells mainly rely on cholesterol absorption [17]. Niemann–Pick C1-like 1 (NPC1L1) plays a central role in the intestinal absorption of cholesterol [81]. Previous studies noticed considerably greater levels of NPC1L1 expression in cancer cells compared to normal cells [82]. Importantly, the upregulation of NPC1L1 is positively associated with tumor progression in various cancers [81,82]. We analyzed the association between the expression of NPC1L1 and progression-free survival (PFS) using publicly available data from a web-based survival analysis tool designed for medical research (KMplot) [83]. Lipid metabolism dysregulation is more common in the advanced stages of OC [10] with a poorer prognosis compared to earlier stages. In stage IV, the cancer has typically spread to organs and tissues in the abdomen, such as the liver, lungs or the lining of the abdomen (peritoneum), or it may have spread to other distant organs such as the spleen or distant lymph nodes [1,2,3].

**Figure 2:**
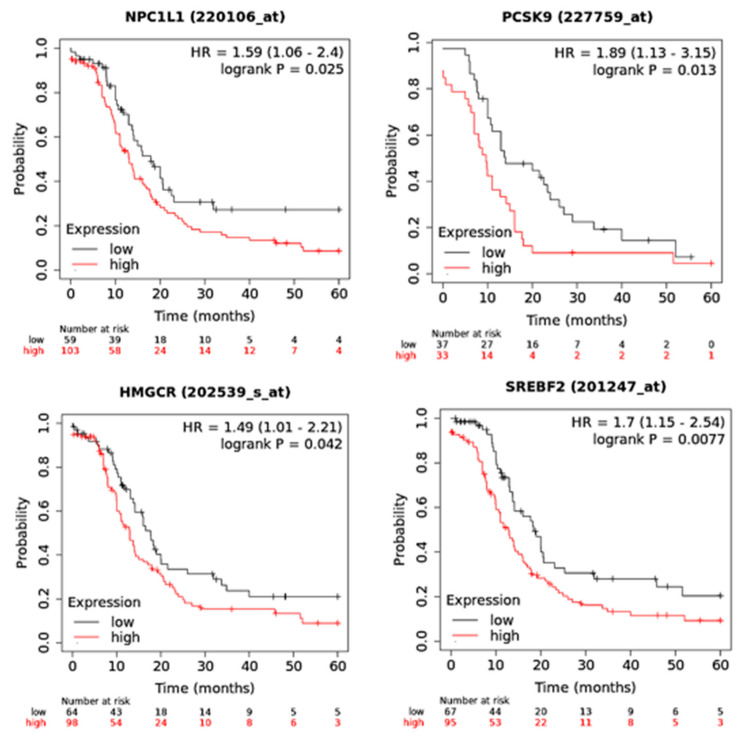
The correlation between PFS and expression levels of NPC1L1, HMGCR, PCSK9 and SREBF2 in stage IV ovarian cancer patients. Kaplan-Meier survival curve plotted for ovarian cancer patients (stage IV) with low or high mean expression of these genes and PFS.

We analyzed the correlation of the abovementioned genes with PFS only in patients with stage IV ovarian cancer, within a 60-month follow-up threshold. Importantly, we noticed significant improvements in PFS in OC patients who expressed lower levels of NPC1L1 (Figure 2). Similar results were demonstrated for PCSK9, HMGCR and SREBF2 in patients with stage IV OC (Figure 2). Collectively, these data suggest the inhibition of cholesterol biosynthesis and absorption as a potential therapeutic strategy in OC.

## 5. Repurposing Hypocholesterolemic Drugs as Anti-Cancer Agents in Ovarian Cancer

Based on evidence of the involvement of aberrant cholesterol metabolism in cancer progression, various researchers are focusing on repurposing existing hypocholesterolemic drugs to treat cancer. Different classes of cholesterol-lowering drugs are available, such as HMG-CoA reductase inhibitors (statins), anti-PCSK9 (alirocumab and evolocumab) and NPC1L1 inhibitors (ezetimibe) [84]. Various studies have investigated the correlation between the usage of statins and mortality in patients with a diagnosis of OC and the majority observed improved overall survival with statin use (Table 1) [85,86,87]. However, the role of statins in OC prevention is controversial. Akinwunmi et al. found a 32% lower risk of OC among women who used statins compared to non-users [88]. Importantly, the highest efficacy of statins was found mainly among those women who used statins for 2 to 4.9 years. In contrast, other studies have suggested that statin usage prior to an OC diagnosis is not correlated with overall survival, and this may be attributable to the presence of comorbidities, which would have a significant impact on the survival rate and treatment outcomes of individuals with cancer [87].

As mentioned above, the upregulation of PCSK9 is one of the main factors that elevates serum cholesterol. Both evolocumab and alirocumab are monoclonal antibodies against PCSK9, which inhibit PCSK9 from binding to the LDLR [40,103,104]. Anti-PCSK9 agents have been used clinically to treat hypercholesterolemic patients [105]. While a few preclinical studies show the efficacy of PCSK9 inhibition in attenuating cell proliferation and survival of ovarian cancer cells [68], the clinical impact of anti-PCSK9 agents is yet to be explored in OC.

Ezetimibe is an inhibitor of NPC1L1 and blocks intestinal cholesterol absorption [106]. Ezetimibe has also been shown to be a potential inhibitor of cancer development and progression by blocking cell proliferation and tumor angiogenesis, as well as the enhancement of the anti-tumor immune response [107,108,109]. Ezetimibe has been proposed as a potent anti-cancer therapeutic agent in a variety of cancers [107], such as pancreatic [110], prostate [108], urinary and bladder [111], breast [112], liver [109], colorectal [113] and melanoma [114]. However, no study has shown the impact of NPC1L1 and ezetimibe in ovarian cancer, and this warrant further investigation.

Altogether, these data suggest that the impact of hypocholesterolemic drugs in ovarian cancer is poorly understood and requires further investigation. It is worth mentioning that other lipid-lowering drug classes, such as fibrates, niacin, bempedoic acid, volanesorsen (anti-apo CIII) and sequestrants including cholestyramine, lodalis, Ethyl eicosapentaenoic acidomega 3, mipomersen and pelacarsen [115,116,117] may warrant investigation as to their lipid-lowering effects on OC progression.

## 6. The Impact of Aberrant Lipid Metabolism on Steroid Hormones in Ovarian Cancer

It has been reported that sexual steroid hormones play a pivotal role in tumor progression in various cancers [118]. In OC, various studies suggest a correlation between the production of sexual steroid hormones and cancer cell proliferation, epithelial-mesenchymal transition (EMT), cell migration, metastasis, apoptosis and resistance to anti-cancer drugs [118,119]. Moreover, the expression of receptors of sexual steroid hormones, including estrogen, progesterone and androgen receptors (ER, PR and AR, respectively), plays an important role in OC progression [8]. OC subtypes display different steroid hormone receptor profiles [118]; for example, the endometrioid subtype is more likely to exhibit estrogen receptor (ER) and/or progesterone receptor (PR) positivity [120], whereas the clear cell carcinoma subtype mainly expresses estrogen receptor beta (Erβ) [121]. Conversely, dysregulated lipid metabolism may also influence the expression and activity of hormone receptors, which potentially modulate the responses of ovarian cancer cells to hormone-based therapies [9].

It Is Important to note that dysregulated lipid metabolism can also impact the production and metabolism of estrogen and progesterone, which may have implications for OC development [8]. Estrogen and progesterone are steroid hormones that play important roles in the regulation of the menstrual cycle, reproductive function and the growth and differentiation of tissues, including the ovaries. In normal physiological conditions, these hormones are tightly regulated, but alterations in lipid metabolism can disrupt their balance and function [120].

Lipids, particularly cholesterol, serve as building blocks for steroid hormones, including estrogen and progesterone. Cholesterol is converted into pregnenolone, which is then further metabolized to produce various steroid hormones, including estrogen and progesterone. Dysregulated lipid metabolism, such as increased cholesterol synthesis or altered cholesterol transport, can influence the availability of cholesterol for hormone synthesis [122]. In OC, dysregulated lipid metabolism can lead to changes in estrogen and progesterone production and metabolism. For example, altered expression or activity of enzymes involved in steroid hormone synthesis pathways can affect the production of estrogen and progesterone. Additionally, dysregulated lipid metabolism can impact the metabolism and clearance of these hormones, potentially leading to elevated hormone levels and increased signaling within cancer cells [9].

Understanding the interplay between dysregulated lipid metabolism, estrogen and progesterone production/metabolism and the response to hormone-based therapies is an active area of research in ovarian cancer that requires further investigation to elucidate any specific association mechanisms and identify potential therapeutic targets, in order to improve treatment outcomes in hormone-dependent ovarian cancer.

## 7. The Role of Aberrant Synthesis of Bile Acids in Ovarian Cancer

As stated above, cholesterol is a critical precursor in the synthesis of various substances, including bile acids [27]. The liver is the primary production site of bile acids, while other tissues can also produce bile acids, such as the ovaries [123]. Emerging evidence suggests a correlation between bile acid levels and cancer, but the impact of bile acids on cancer progression remains controversial [123]. Bile acids have been reported as a tumor promoter in various cancers, such as colorectal, breast and prostate [124], while other studies suggest bile acids as therapeutic agents [125]. Horowitz et al. found that deoxycholic acid and ursodeoxycholic acid administration resulted in statistically significant dose-dependent cytotoxicity in both platinum-sensitive and platinum-resistant cell lines via apoptosis [126]. Jin et al. [127] identified the downregulation of estrogen receptor 1 (ER1) in ovarian cancer cells upon treatment with bile acids, suggesting a therapeutic effect, given that upregulated ER is positively associated with peritoneal metastasis in endometrioid ovarian cancer [128]. In summary, further investigation is required to identify the role of bile acids in ovarian cancer.

## 8. Conclusions

The relationship between lipid pathways and ovarian cancer is an area of ongoing research. Lipids play important roles in various cellular processes, including cell signaling, energy storage and membrane structure. The dysregulation of lipid metabolism has been implicated in the development and progression of various cancers, including ovarian cancer. It is important to note that the understanding of the specific mechanisms linking lipid pathways to ovarian carcinogenesis is still evolving, and more research is needed to fully elucidate their roles. The complex interplay between lipid metabolism and ovarian cancer involves multiple factors and pathways, and aberrant cholesterol metabolism appears to be an essential factor in ovarian cancer progression. Therefore, further studies are required to fully understand the impact of cholesterol on ovarian cancer and to elucidate the involved mechanistic pathways that modulate cell proliferation, metastasis and drug resistance. Understanding of the various target pathways will form the foundation of the investigation into hypocholesterolemic drugs and provide a pivotal therapeutic approach to slow the progression of ovarian cancer.

## Figures and Tables

**Table 1:** Hypocholesterolemic drugs’ effects on ovarian cancer [68,86,87,89,90,91,92,93,94,95,96,97,98,99,100,101,102].

Drug	Mechanism of Action	Studies	Studies Type	Observation
Statins	Blocking hepatic HMGCR, rate limiting step in Cholesterol synthesis	Elmore RG (2008) [89] Nielsen SF (2012) [90] Lavie O (2013) [91] Habis M (2014) [92] Bar D (2016) [93] Chen HY (2016) [94] Wang A (2016) [95] Couttenier A (2017) [96] Verdoodt F (2017) [97] Vogel TJ (2017) [98] Urpilainen E (2018) [99] Harding BN (2019) [100] Feng JL (2021) [87] Hanley GE (2021) [101] Kim DS (2021) [102] Majidi A (2021) [86]	Clinical	Improve Overall Survival time. (HR, 0.79; 95% CI, 0.73–0.85; *p* < 0.00001)
PCSK9 Inhibitor	Inhibit PCSK9 enzyme, ↓ LDLR	Sanz DJ (2021) [68]	Preclinical (OC cell lines)	Impairs cancer cell growth

## Data Availability

RNA-seq data are publicly available in Kaplan-Meier plotter website with the accession 220106-at, 227759-at, 202539-at, and 201247-at for NPC1L1, PCSK9, HMGCR, and SREBF2, respectively.
